# Regional variation and outcome of out-of-hospital cardiac arrest (ohca) in Finland – the Finnresusci study

**DOI:** 10.1186/1757-7241-20-80

**Published:** 2012-12-17

**Authors:** Pamela Hiltunen, Markku Kuisma, Tom Silfvast, Juha Rutanen, Jukka Vaahersalo, Jouni Kurola

**Affiliations:** 1 Department of Prehospital Emergency Care, Emergency and Intensive Care, Kuopio University Hospital, Kuopio, Finland; 2 EMS, department of anaesthesia and ICM, Helsinki University Central Hospital, Helsinki, Finland; 3 Department of Intensive Care, Helsinki University Central Hospital, Helsinki, Finland

## Abstract

**Background:**

Despite the efforts of the modern Emergency Medical Service Systems (EMS), survival rates for sudden out-of-hospital cardiac arrest (OHCA) have been poor as approximately 10% of OHCA patients survive hospital discharge. Many aspects of OHCA have been studied, but few previous reports on OHCA have documented the variation between different sizes of study areas on a regional scale. The aim of this study was to report the incidence, outcomes and regional variation of OHCA in the Finnish population.

**Methods:**

From March 1st to August 31st, 2010, data on all OHCA patients in the southern, central and eastern parts of Finland was collected. Data collection was initiated via dispatch centres whenever there was a suspected OHCA case or if a patient developed OHCA before arriving at the hospital. The study area includes 49% of the Finnish population; they are served by eight dispatch centres, two university hospitals and six central hospitals.

**Results:**

The study period included 1042 cases of OHCA. Resuscitation was attempted on 671 patients (64.4%), an incidence of 51/100,000 inhabitants/year. The initial rhythm was shockable for 211 patients (31.4%). The survival rate at one-year post-OHCA was 13.4%. Of the witnessed OHCA events with a shockable rhythm of presumed cardiac origin (n=140), 64 patients (45.7%) were alive at hospital discharge and 47 (33.6%) were still living one year hence. Surviving until hospital admission was more likely if the OHCA occurred in an urban municipality (41.5%, p=0.001).

**Conclusions:**

The results of this comprehensive regional study of OHCA in Finland seem comparable to those previously reported in other countries. The survival of witnessed OHCA events with shockable initial rhythms has improved in urban Finland in recent decades.

## Background

Sudden out-of-hospital cardiac arrest (OHCA) is a major public health concern [1]. The overall incidence of OHCA is reportedly between 37 and 121 per 100,000 inhabitants/year, and the overall survival rate to hospital discharge varies from 4.5 to 10.7% [2-7]. It is notable that the definitions of incidence and outcome of OHCA vary across reports [8], complicating study comparison.

It has previously been estimated that the survival of OHCA with a shockable initial rhythm decreases by 10% for every minute that cardiopulmonary resuscitation (CPR) is delayed [9]. The most important factors affecting survival are whether or not the OHCA is witnessed, how fast CPR is started and whether or not the initial rhythm is shockable) [4,10-12].

Previous studies, conducted in urban centres in Finland, have reported the incidence of OHCA as 46–80/100,000 inhabitants per year and the survival to hospital discharge between 13–27% [11,13,14]. There are no reports from Finland regarding incidence, EMS delay or outcomes of OHCA in neither semi-urban nor rural areas. Therefore, the existence of regional variation is unknown. To our knowledge, few previous reports discuss OHCA with a regional perspective [5,15,16].

The aim of this study was to determine the incidence and outcomes of OHCA in the urban, semi-urban and rural Finnish populations. In addition, we studied whether the outcomes for urban patients with shockable rhythms have improved since previous reports.

## Methods

### Study design

This study was a prospective observational cohort study conducted in southern and eastern Finland from March 1^st^, 2010, to August 31^st^, 2010. It included all patients in the study areas who fulfilled the criteria of suspected OHCA according to uniform national emergency medical dispatch policy. Furthermore, all patients who were identified as having suffered from OHCA by EMS crews or who developed OHCA before arrival at the hospital were included. The study protocol was approved by the Institutional Review Board of the Helsinki University Central Hospital.

### Study area and population

**Figure 1:**
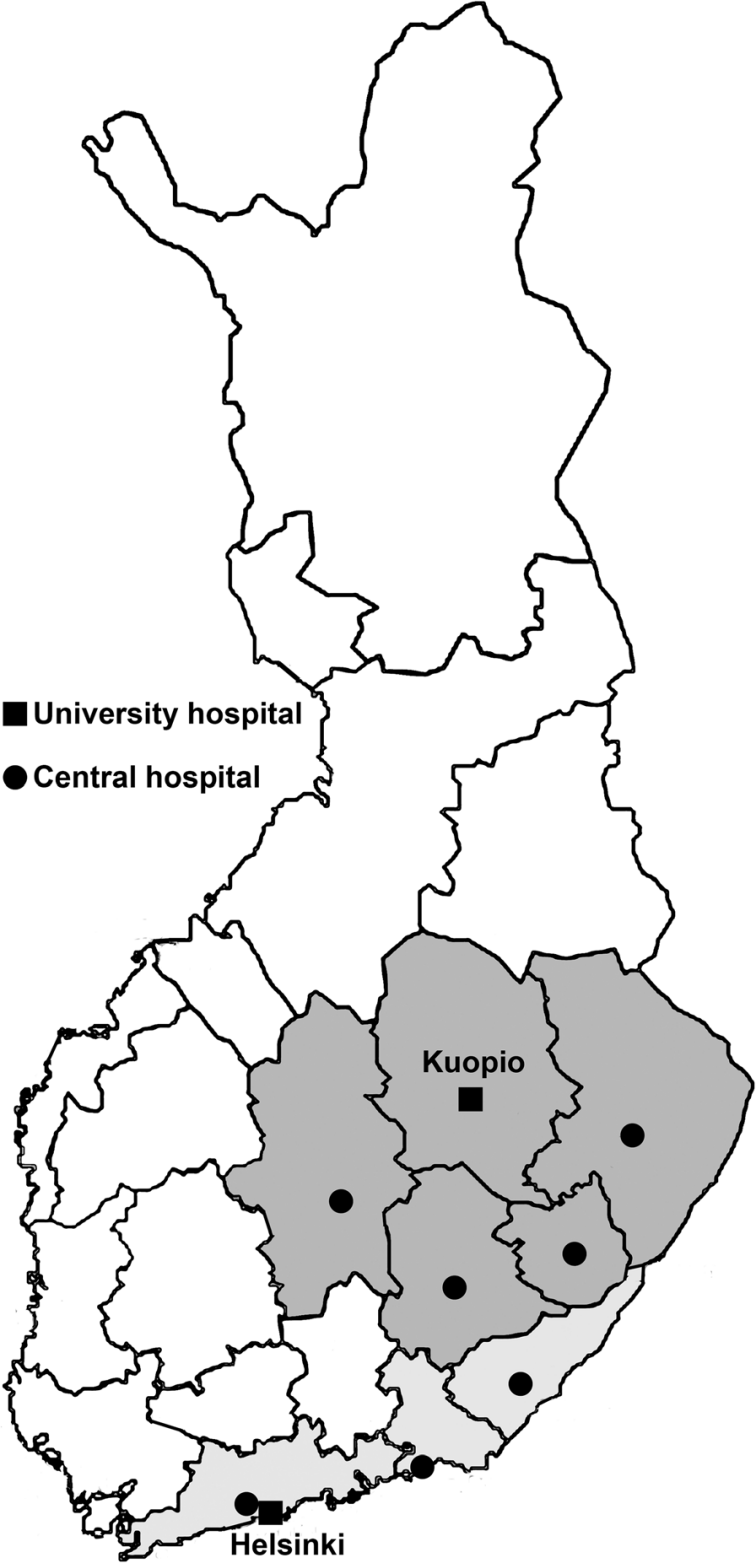
Finnresusci study area.

Finland is the most sparsely populated country in the European Union (geographic area 337,000 km^2^, population density 17/km^2^). The overall population included in this study was 2,644,200 (49.1% of the total Finnish population). Figure 1 shows the study area with its two university and six central hospitals, as well as Helsinki, the Finnish capital.

The Finnresusci study area covered 120 municipalities. For the purpose of this study, the municipalities were divided into urban, semi-urban and rural [17]. In urban municipalities, over 90% of the population lives in densely populated communities, and the largest community exceeds 15,000 residents. In semi-urban municipalities, the corresponding figures are 60–90% and 4,000-14,000 residents, respectively. In rural municipalities, less than 60% live in densely populated communities, and the size of the most densely populated community is less than 4,000 residents.

### Description of the EMS system

Eight regional dispatch centres are located within the study area. Trained dispatchers process emergency calls. The centres combine EMS, police, and fire and rescue services, and they are connected to a common database.

EMS services are three-tiered: trained first-responders serve as the first tier, the second tier consists of advanced life-support units with paramedics, and the third tier is a prehospital emergency physician staffed unit.

Guidelines defining the treatment of OHCA in Finland [18] conform to the guidelines issued by the European Resuscitation Council (ERC) [19]. For patients with non-witnessed OHCA and primary asystole rhythms, or those for whom OHCA is followed by an evidently fatal event, resuscitation attempts can be withheld. Paramedics can decide to withhold resuscitation according to the national guideline, DNAR (do not attempt resuscitation) orders and/or online consultation with a prehospital emergency physician.

### Data collection and analysis

In order to provide a uniform database for this study, as well as to ensure the inclusion of all patients in the study area, an interface was created between the dispatch centres’ combined databases and the database of the Finnish Quality Consortium of Intensive Care [20].

The EMS crew collected data on patients who met the inclusion criteria and faxed the case report forms (CRF) to a research nurse who entered the data into the common Finnresusci database and linked them to the dispatch data. The principal investigator received information about patients’ status at the time of hospital discharge from the National Institute for Health and Welfare, and data regarding OHCA survival after one year was obtained from the Finnish Population Information System. The population used in this study was announced from this registry on December 31^st^, 2009 [21].

For the purpose of this study, we classified a patient as **considered** for resuscitation unless the patient was alive upon EMS arrival despite being dispatched as a case of cardiac arrest, or unless the patient had secondary signs of death. The definition “considered for resuscitation” was used only to calculate the incidence of these cases. Resuscitation was defined as **attempted** unless the EMS crew immediately discontinued basic CPR after initial assessment due to the considered futility of the situation (i.e., unwitnessed arrest and asystole), or because of a pre-existing DNAR order.

The basic timeline covered the collapse time estimated by EMS personnel from the start of chest compressions (from a bystander or EMS) to the time of first defibrillation.

Outcome was determined by the rate of survival at hospital discharge and at one year post OHCA divided by the number of patients with **attempted** resuscitation. We considered patients discharged when they were transferred from their primary hospital to a non-acute care facility or discharged home.

Data were analyzed using SPSS version 17.0 (SPSS, Chicago, Ill., USA). Data are presented as medians with IQR or as frequencies and percentages. Association between categorical variables was assessed using cross tabulation and the chi-square test, and the variables were given in time points according to the Kruskal-Wallis one-way analysis of variance. The Kaplan-Meier estimator was used to assess the difference in patient survival between the municipalities (one-year follow-up). A *p*-value less than 0.05 was considered statistically significant. We used a logistic regression model in order to find factors related to survival at one year. Age was a dichotomous variable (under 18, 18–45, 45–65 and over 65 years of age) and time from collapse to CPR was a continuous variable.

### Outcome tracking

The end points of the study were survival to hospital discharge and survival status after one year.

## Results

**Figure 2:**
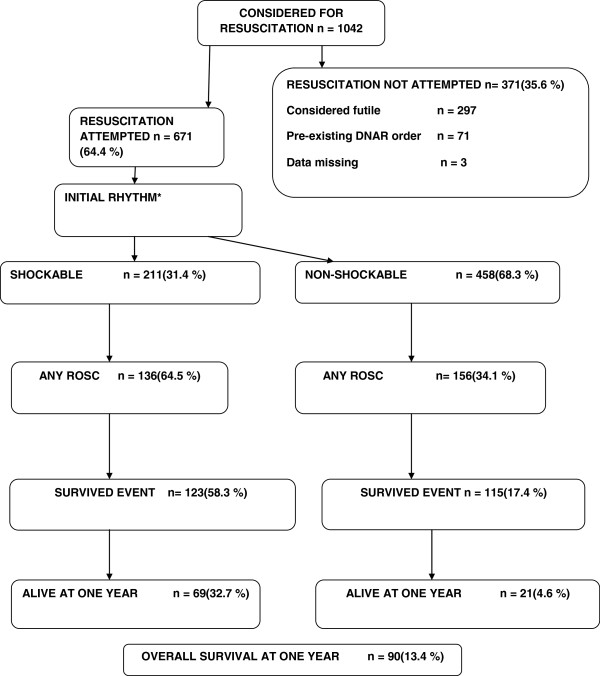
**Study flow chart.** Out-of-hospital cardiac arrests in Finland, considered, attempted, not attempted and outcome. * In two cases the initial rhythm were not monitored.

During the study period, EMS **considered** resuscitation on 1042 patients, a corresponding incidence of 78/100,000 inhabitants/year. The reasons for refraining from resuscitation are presented in Figure 2. Resuscitation was **attempted** for 671 patients (64.4%). The final analysis covered this subgroup.

The incidence of attempted resuscitation was 51/100,000/inhabitants/year. The baseline characteristics of these patients are presented in Table 1, and the time intervals are presented in Table 2. The initial rhythm was shockable for 211 patients (31.4% of patients on whom resuscitation was attempted).

**Table 1:** Baseline characteristics of patients in final analysis

**Variable**	**n=671 (%)**
**Sex**	
Male	475(70.8)
Female	196(29.2)
**Shockable rhythm**	211(31.4)
**Age (median)**	66(IQR56-78)
**Location of arrest**	
Home	398(59.3)
Public	152(22.7)
Health care emergency	16(2.4)
Extented care facility	63(9.4)
Ambulance	41(6.1)
Data missing	1(0.1)
**Municipality**	
Urban	410(61.1)
Semi urban	106(15.8)
Rural	155(23.1)
**Presumed etiology**	
Cardiac	361(53.8)
Unknown	156(23.2)
Hypoxia	62(9.2)
Submersion	24(3.6)
Exsanguination	15(2.2)
Trauma	10(1.5)
Intoxication	8(1.2)
Electrocution	3(0.4)
Hypothermia	2(0.3)
Other	26(3.9)
Data missing	4(0.6)
**Witnessed**	
By close relative/layperson	459(68.4)
By EMS	140(20.9)
Not witnessed	60(8.9)
Data missing	12(1.8)
**Adrenalin given during CPR**	488(72.7)

**Table 2:** Time intervals in minutes (median (IQR)) in municipalities from collapse to cardiopulmonary resuscitation* (CPR) given by bystander or Emergency Medical Service (EMS) and from collapse to defibrillation in patients with initial shockable rhythm

**Type of municipality**	Time from collapse to CPR*	**Time from collapse to defibrillation in initial shockable rhythm**
**Urban**	3 (0–8)	9,5 (6–13)
**Semi urban**	2 (0–10)	10,5 (7,75-16,5)
**Rural**	3 (0–10)	12 (5–20,5)
**Study area**	3 (0–9)	10 (6–14)
**P-value**	0.884	0.209

P-values are counted in Kruskal-Wallis test.

For resuscitated patients, approximately half (47.2%) received at least bystander chest compressions or standard CPR before EMS arrival. The cause of arrest was presumed to be of cardiac origin for 361 patients (53.8%). Any degree of return of spontaneous circulation (ROSC) was achieved on the scene for 294 patients (43.8%).

In all, 133 patients (19.9%) survived until hospital discharge. Thirty-three of them were discharged home and 100 were discharged to a non-acute ward or facility. One year later, 90 of the discharged patients (13.4%) were still living.

Of the 140 patients who suffered a bystander-witnessed arrest (i.e., not witnessed by EMS) with shockable rhythm due to a condition of presumed cardiac origin, 64 (45.7%) were alive at hospital discharge, and 47 (33.6%) were still alive after one year. In urban areas, the numbers were 53.8% and 40.9%, respectively.

Of the 671 patients, 140 suffered an EMS-witnessed OHCA. Of those patients, 107 (76.4%) exhibited a non-shockable rhythm. The numbers of EMS-witnessed OHCA patients who survived until hospital discharge and at one year were 36 (25.7%) and 23 (16.4%), respectively.

EMS crews attempted resuscitation in 63 cases when the location of arrest was in an extended care facility. Four of these patients (6.3%) were still alive one year later. OHCA was of traumatic origin for 10 patients. One patient survived until hospital admission but died later the same day in an intensive care unit.

The three elements of resuscitation attempts, initial rhythm and survival are presented in relation to municipality type in Table 3. In urban municipalities, more patients survived until hospital admission (41.5%) than in semi-urban (28.3%) or rural (25.8%) municipalities (p=0.001).

**Table 3:** Type of municipality and resuscitation attempts, initial rhythms and outcome

**Type of municipality**	**Resuscitation attempted (%)**	Initial rhythm (%)*	**Admitted to hospital (%)**	**Alive at hospital discharge (%)**	Alive at one year (%)**
**Urban**	n=410 (63.9)	**Shockable**	n=170(41.5)	n=97(23.7)	n=62(15.1)
		n=137(33.4)			
		**Non-shockable**			
		n=271(66.1)			
**Semi urban**	n=106 (63.9)	**Shockable**	n=30(28.3)	n=18(17.0)	n=14(13.2)
		n=34(32.1)			
		**Non-shockable**			
		n=72(67.9)			
**Rural**	n=155 (66.2)	**Shockable**	n=40(25.8)	n=18(11.6)	n=14(9.0)
		n=40(25.8)			
		**Non-shockable**			
		n=115(74.2)			
**Study area**	n=671 (64.4)	**Shockable**	n=240(35.8)	n=133(20.3)	n=90(13.4)
		n=211(31.4)			
		**Non-shockable**			
		n=458(68.3)			
**P**	p=0.800	p=0.232	p=0.001	p=0.344	p=0.153

P-values are counted in chi-square-test.

*in two cases the initial rhythm were not monitored.

** Survival after one year could not be established in four patients who were discharged alive from hospital to their home countries.

**Figure 3:**
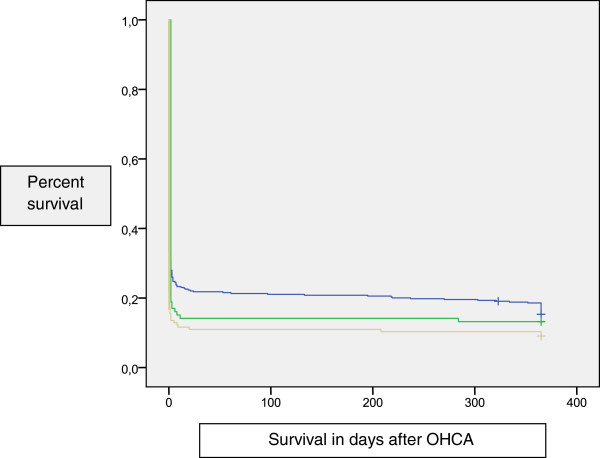
**Kaplan-Meier survival analysis.** Difference between municipalities and survival (one year follow-up). Municipalities: Blue: Urban Green: Semi-urban Yellow: Rural.

Outcomes at one year were 6.1% greater in urban than in rural areas (CI −0.05 to 12.2), 4.2% higher in semi-urban than in rural areas (CI −12.8 to 4.5) and 1.9% higher in urban than in semi-urban areas (CI −9.8 to 6.0). However, these differences were not statistically significant. Figure 3 shows the survival analysis (using Kaplan Meier) in different municipalities at the one-year follow-up.

In multivariate analysis, shockable initial rhythm (p=0.000, OR 6.65, 95% CI 3.56 to 12.4), short delay from collapse to the beginning of CPR (p=0.001, OR 0.86, 95% CI 0.79 to 0.94), arrest presumed to be of cardiac origin (p=0.05, OR 2.0, 95% CI 1.06 to 3.9) and age (p=0.000, OR 0.97, 95% CI 0.95 to 0.98) were related to survival at one year. Other variables tested in this model were municipality type, gender, whether OHCA was witnessed (not by EMS) or not, EMS-witnessed OHCA and location of OHCA, but these variables were not statistically significant. In a subgroup with shockable initial rhythm, the same multivariate logistic regression model showed that cause of presumed cardiac origin (p=0.012, OR 6.18, 95% CI 1.50-25.5), short time delay between collapse and first defibrillation (p=0.033, OR 0.87, 95% CI 0.77-0.99) and young age (p=0.002, OR 0.95, 95% CI 0.92-0.98) were independent factors related to survival at one year.

## Discussion

This is the first study of OCHA to cover nearly half of the Finnish population. The main findings were: 1) the incidence of attempted resuscitation in Finland was 51/100,000 inhabitants/year, and survival from all events and rhythms one year after the incident was 13.4%; 2) the initial rhythm was shockable in 31.4% of patients, and 32.7% of those patients were alive after one year; and 3) 53.8% of the OHCA patients in urban areas survived until hospital discharge when their cardiac arrest was witnessed, their initial rhythm was shockable and their cause of arrest was presumed to be of cardiac origin. This result seems to show an improvement over previous reports [14,22-24]. In a study by Silfvast in 1990 on patients in the capital city of Helsinki, the survival of these patients at hospital discharge was 27% [13]. Their survival rate rose to 32.5% in 1994 [14]. In another Finnish report on the city of Tampere in 2007, the survival in this subgroup was 28% [11]. It seems that the prognosis of these patients has improved in Finland, and similar improvement has been demonstrated in reports from other countries [5,24,25].

The survival rate for patients who experienced OHCA with all rhythms and in all situations at one year was 13.4%. This can be considered an acceptable outcome because the study area was wide and included rural municipalities as well as urban and semi-urban areas. In a randomized trial of intravenous epinephrine in non-traumatic, all-rhythm-OHCA in Norway, the survival to hospital discharge was 9.2% for the epinephrine-treated group and 10.5% for the non-treated group [6]. In another study from Sweden, the one-month survival of patients with all rhythms varied between 2% and 14% [15].

Therapeutic hypothermia (TH) has been shown to improve the outcomes of resuscitated patients [26,27]. The use of TH after OCHA [28] is the standard of care in Finnish intensive care units [29,30] and might be one explanation for better survival compared to previous reports. In addition, the national guidelines [18] and protocols for the treatment of OCHA patients are likely to guide the overall treatment towards more standardized methods for the EMS and in ICUs [28]. Finally, the ‘chain of survival’ in OHCA patients is dictated by the prompt recognition of cardiac arrest (by both the bystander and the EMS dispatcher) and by the rapid response of the EMS. The dispatcher can play a major role in guiding a bystander to start CPR. In a Finnish study, 83% of OHCA patients were adequately recognized by the dispatchers [31].

Of the patients with non-shockable rhythms, 21 (4.6%) were alive after one year. Similar results have been reported by other investigators [10]. Although this survival rate is modest, this group should be kept in mind when treating OHCA patients, especially when there is a short delay in the administration of advanced life support and achieving ROSC [12,32].

In the current study, the presence of EMS at the moment of collapse had no effect on survival at one year (p=0.198). This is in agreement with two previous studies that also document that EMS-witnessed OHCA patients usually exhibit a non-shockable rhythm [33,34]. The administration of CPR before EMS arrival did not positively affect survival at one year in the current study. In previous studies, bystander CPR has been found to be a significant factor for in survival [14,35]. Our result may be due to lack of statistical power. In addition, less than half (47.2%) of the OHCA patients in the present study received CPR overall.

Survival to hospital admission was associated with municipality type. The patients in urban municipalities survived to hospital admission more often than patients in the other two municipality types. There was no difference in the distribution of primary rhythms between the municipalities. It is difficult to explain factors related to the observed difference in survival to hospital admission. There was no statistical difference between municipalities in survival rates at one year. This could be explained by a lack of power. Still, there was a clinically meaningful difference in survival between urban (15.1%) and rural regions (9.0%).

As previously shown [35], a short delay from collapse to the beginning of CPR (whether by bystander or EMS) was an independent factor of survival at one year (p=0.001), and a quick response with the first defibrillation after collapse (p=0.003). There was a trend for longer delays between urban and rural municipalities, but this difference was not statistically significant.

Researchers should discuss further how to report the incidence of OHCA in response to the lack of definitions for OHCA and who is “considered for resuscitation”. Despite the Utstein template [36], there is still great variation among reports on OHCA [1,22,37]. Previous reports on OHCA have included all patients who died suddenly, those for whom resuscitation efforts were attempted, and patients who fell in between these two states [8]. We suggest that all OHCA patients encountered by the EMS should be included in the calculation of the **incidence** of OHCA. All patients not obviously dead (e.g., decapitated or with secondary signs of death) should be reported as **considered** for resuscitation. Of the patients considered for resuscitation, only those for whom resuscitation was **attempted** should then form the basis for reporting the outcomes of OHCA, while recording a different dataset of those in whom resuscitation was not initiated. The outcome numbers have been calculated in this manner in some previous reports [5,7,14,38].

There were some limitations to this study. Some time points were estimates by the EMS crews, the accuracy of which could be affected by the fact that differences in time have previously been documented between the clocks at dispatch centres and the clocks on defibrillators [39]. Because our study design included all patients who developed OHCA (i.e., in the presence of EMS personnel), we did not always have the time of the beginning of the dispatch call and we therefore had to rely on the EMS crews’ reports of time points.

Furthermore, we lost the data on four patients in the follow-up. Data on the initial rhythm were missing for two patients. Despite close follow-up and frequent feedback, we cannot be certain that every single cardiac arrest occurrence in the study area was included.

## Conclusions

This first comprehensive study of OHCA covering half of the population of Finland included all types of geographic areas. It revealed that the incidence of OHCA with attempted resuscitation was 51/100,000 inhabitants/year and that the overall survival one year after OHCA was 13.4%. Survival until hospital admission was most likely in urban municipalities (p=0.001). In urban Finnish regions, the survival rate from witnessed OHCA events for people with shockable initial rhythms has improved compared with previous reports.

## Competing interests

The authors declare that they have no competing interests.

## Authors’ contributions

The study protocol was discussed, written and edited by all authors. All participated collecting the data. PH analyzed the data with JK. The manuscript was drafted by PH and all authors contributed to the editing and accepted the final version of the article.

## Financial support

This research was supported by the EVO funding of Kuopio University Hospital, by Foundation of Emergency Medicine, by Finska Läkaresällskapet Foundation and by the Päivikki and Sakari Sohlberg Foundation (http://www.pss-saatio.fi). The study sponsors had no involvement in the study design, in the collection, analysis and interpretation of data, in the writing of the manuscript or in the decision to submit the manuscript for publication.

## References

[B1] Atwood C Eisenberg MS Herlitz J Rea TD Incidence of EMS-treated out-of-hospital cardiac arrest in Europe Resuscitation 2005 67 1 75 80 10.1016/j.resuscitation.2005.03.021 16199289

[B2] Rea TD Eisenberg MS Sinibaldi G White RD Incidence of EMS-treated out-of-hospital cardiac arrest in the United States Resuscitation 2004 63 1 17 24 10.1016/j.resuscitation.2004.03.025 15451582

[B3] de Vreede-Swagemakers JJ Gorgels AP Dubois-Arbouw WI van Ree JW Daemen MJ Houben LG Wellens HJ Out-of-hospital cardiac arrest in the 1990's: a population-based study in the Maastricht area on incidence, characteristics and survival J Am Coll Cardiol 1997 30 6 1500 1505 10.1016/S0735-1097(97)00355-0 9362408

[B4] Cobb LA Fahrenbruch CE Olsufka M Copass MK Changing incidence of out-of-hospital ventricular fibrillation, 1980–2000 JAMA 2002 288 23 3008 3013 10.1001/jama.288.23.3008 12479765

[B5] Nichol G Thomas E Callaway CW Hedges J Powell JL Aufderheide TP Rea T Lowe R Brown T Dreyer J Davis D Idris A Stiell I Resuscitation Outcomes Consortium Investigators Regional variation in out-of-hospital cardiac arrest incidence and outcome JAMA 2008 300 12 1423 1431 10.1001/jama.300.12.1423 18812533 PMC3187919

[B6] Olasveengen TM Sunde K Brunborg C Thowsen J Steen PA Wik L Intravenous drug administration during out-of-hospital cardiac arrest: a randomized trial JAMA 2009 302 20 2222 2229 10.1001/jama.2009.1729 19934423

[B7] Lindner TW Soreide E Nilsen OB Torunn MW Lossius HM Good outcome in every fourth resuscitation attempt is achievable-An Utstein template report from the Stavanger region Resuscitation 2011 82 12 1508 1513 10.1016/j.resuscitation.2011.06.016 21752524

[B8] Berdowski J Berg RA Tijssen JG Koster RW Global incidences of out-of-hospital cardiac arrest and survival rates: Systematic review of 67 prospective studies Resuscitation 2010 81 11 1479 1487 10.1016/j.resuscitation.2010.08.006 20828914

[B9] Cummins RO Ornato JP Thies WH Pepe PE Improving survival from sudden cardiac arrest: the "chain of survival" concept. A statement for health professionals from the Advanced Cardiac Life Support Subcommittee and the Emergency Cardiac Care Committee, American Heart Association Circulation 1991 83 5 1832 1847 10.1161/01.CIR.83.5.1832 2022039

[B10] Holmgren C Bergfeldt L Edvardsson N Karlsson T Lindqvist J Silfverstolpe J Svensson L Herlitz J Analysis of initial rhythm, witnessed status and delay to treatment among survivors of out-of-hospital cardiac arrest in Sweden Heart 2010 96 22 1826 1830 10.1136/hrt.2010.198325 20889992

[B11] Kamarainen A Virkkunen I Yli-Hankala A Silfvast T Presumed futility in paramedic-treated out-of-hospital cardiac arrest: an Utstein style analysis in Tampere, Finland Resuscitation 2007 75 2 235 243 10.1016/j.resuscitation.2007.04.011 17553611

[B12] Herlitz J Svensson L Engdahl J Silfverstolpe J Characteristics and outcome in out-of-hospital cardiac arrest when patients are found in a non-shockable rhythm Resuscitation 2008 76 1 31 36 10.1016/j.resuscitation.2007.06.027 17709164

[B13] Silfvast T Prehospital resuscitation in Helsinki, Finland Am J Emerg Med 1990 8 4 359 364 10.1016/0735-6757(90)90097-J 2363761

[B14] Kuisma M Maatta T Out-of-hospital cardiac arrests in Helsinki: Utstein style reporting Heart 1996 76 1 18 23 10.1136/hrt.76.1.18 8774321 PMC484418

[B15] Stromsoe A Svensson L Claesson A Lindkvist J Lundstrom A Herlitz J Association between population density and reported incidence, characteristics and outcome after out-of-hospital cardiac arrest in Sweden Resuscitation 2011 82 10 1307 1313 10.1016/j.resuscitation.2011.04.025 21628082

[B16] Wang HE Devlin SM Sears GK Vaillancourt C Morrison LJ Weisfeldt M Callaway CW the ROC Investigators Regional variations in early and late survival after out-of-hospital cardiac arrest Resuscitation 2012 83 11 1343 1348 10.1016/j.resuscitation.2012.07.013 22824170 PMC3470746

[B17] Municipalities divided as urban, semi-urban and rural 2012 [http://www.stat.fi/meta/luokitukset/kunta/001-2012/luokitusavain_kuntar.html]

[B18] Suomalaisen Laakariseuran Duodecimin, Suomen Elvytysneuvoston, Suomen Anestesiologiyhdistyksen, Suomen Punaisen Ristin Asettama Tyoryhma Update on current care guidelines: resuscitation Duodecim 2011 127 10 1061 1063 21696005

[B19] Nolan JP Soar J Zideman DA Biarent D Bossaert LL Deakin C Koster RW Wyllie J Bottiger B ERC Guidelines Writing Group European Resuscitation Council Guidelines for Resuscitation 2010 Section 1 Executive summary. Resuscitation 2010 81 10 1219 1276 10.1016/j.resuscitation.2010.08.021 20956052

[B20] Intensium website[http://www.intensium.fi/web/english/]

[B21] Population Register Centre [http://pxweb2.stat.fi/database/StatFin/vrm/vaerak/vaerak_fi.asp]

[B22] Franek O Pokorna M Sukupova P Pre-hospital cardiac arrest in Prague, Czech Republic–the Utstein-style report Resuscitation 2010 81 7 831 835 10.1016/j.resuscitation.2010.03.005 20413205

[B23] Fischer M Fischer NJ Schuttler J One-year survival after out-of-hospital cardiac arrest in Bonn city: outcome report according to the 'Utstein style' Resuscitation 1997 33 3 233 243 10.1016/S0300-9572(96)01022-2 9044496

[B24] Adielsson A Hollenberg J Karlsson T Lindqvist J Lundin S Silfverstolpe J Svensson L Herlitz J Increase in survival and bystander CPR in out-of-hospital shockable arrhythmia: bystander CPR and female gender are predictors of improved outcome. Experiences from Sweden in an 18-year perspective Heart 2011 97 17 1391 1396 10.1136/hrt.2011.222711 21715444

[B25] Lund-Kordahl I Olasveengen TM Lorem T Samdal M Wik L Sunde K Improving outcome after out-of-hospital cardiac arrest by strengthening weak links of the local Chain of Survival; quality of advanced life support and post-resuscitation care Resuscitation 2010 81 4 422 426 10.1016/j.resuscitation.2009.12.020 20122786

[B26] Bernard SA Gray TW Buist MD Jones BM Silvester W Gutteridge G Smith K Treatment of comatose survivors of out-of-hospital cardiac arrest with induced hypothermia N Engl J Med 2002 346 8 557 563 10.1056/NEJMoa003289 11856794

[B27] Hypothermia after Cardiac Arrest Study Group Mild therapeutic hypothermia to improve the neurologic outcome after cardiac arrest N Engl J Med 2002 346 8 549 556 11856793 10.1056/NEJMoa012689

[B28] Castren M Silfvast T Rubertsson S Niskanen M Valsson F Wanscher M Sunde K Task Force on Scandinavian Therapeutic Hypothermia Guidelines, Clinical Practice Committee Scandinavian Society of Anaesthesiology and Intensive care Medicine Scandinavian clinical practice guidelines for therapeutic hypothermia and post-resuscitation care after cardiac arrest Acta Anaesthesiol Scand 2009 53 3 280 288 10.1111/j.1399-6576.2008.01881.x 19243313

[B29] Oksanen T Pettila V Hynynen M Varpula T Intensium Consortium study group Therapeutic hypothermia after cardiac arrest: implementation and outcome in Finnish intensive care units Acta Anaesthesiol Scand 2007 51 7 866 871 10.1111/j.1399-6576.2007.01365.x 17635393

[B30] Reinikainen M Oksanen T Leppanen P Torppa T Niskanen M Kurola J Finnish Intensive Care Consortium Mortality in out-of-hospital cardiac arrest patients has decreased in the era of therapeutic hypothermia Acta Anaesthesiol Scand 2012 56 1 110 115 10.1111/j.1399-6576.2011.02543.x 22091826

[B31] Nurmi J Pettila V Biber B Kuisma M Komulainen R Castren M Effect of protocol compliance to cardiac arrest identification by emergency medical dispatchers Resuscitation 2006 70 3 463 469 10.1016/j.resuscitation.2006.01.016 16870317

[B32] Vayrynen T Kuisma M Maatta T Boyd J Who survives from out-of-hospital pulseless electrical activity? Resuscitation 2008 76 2 207 213 10.1016/j.resuscitation.2007.07.023 17804144

[B33] Hostler D Thomas EG Emerson SS Christenson J Stiell IG Rittenberger JC Gorman KR Bigham BL Callaway CW Vilke GM Beaudoin T Cheskes S Craig A Davis DP Reed A Idris A Nichol G Resuscitation Outcomes Consortium Investigators Increased survival after EMS witnessed cardiac arrest. Observations from the Resuscitation Outcomes Consortium (ROC) Epistry-Cardiac arrest Resuscitation 2010 81 7 826 830 10.1016/j.resuscitation.2010.02.005 20403656 PMC2893256

[B34] Kuisma M Maatta T Repo J Cardiac arrests witnessed by EMS personnel in a multitiered system: epidemiology and outcome Am J Emerg Med 1998 16 1 12 16 10.1016/S0735-6757(98)90058-4 9451307

[B35] Sasson C Rogers MA Dahl J Kellermann AL Predictors of survival from out-of-hospital cardiac arrest: a systematic review and meta-analysis Circ Cardiovasc Qual Outcomes 2010 3 1 63 81 10.1161/CIRCOUTCOMES.109.889576 20123673

[B36] Jacobs I Nadkarni V Bahr J Berg RA Billi JE Bossaert L Cassan P Coovadia A D'Este K Finn J Halperin H Handley A Herlitz J Hickey R Idris A Kloeck W Larkin GL Mancini ME Mason P Mears G Monsieurs K Montgomery W Morley P Nichol G Nolan J Okada K Perlman J Shuster M Steen PA Sterz F Tibballs J Timerman S Truitt T Zideman D International Liason Committee on Resusitation Cardiac arrest and cardiopulmonary resuscitation outcome reports: update and simplification of the Utstein templates for resuscitation registries. A statement for healthcare professionals from a task force of the international liaison committee on resuscitation (American Heart Association, European Resuscitation Council, Australian Resuscitation Council, New Zealand Resuscitation Council, Heart and Stroke Foundation of Canada, InterAmerican Heart Foundation, Resuscitation Council of Southern Africa) Resuscitation 2004 63 3 233 249 10.1016/j.resuscitation.2004.09.008 15582757

[B37] Holmberg M Holmberg S Herlitz J Gardelov B Survival after cardiac arrest outside hospital in Sweden Swedish Cardiac Arrest Registry. Resuscitation 1998 36 1 29 36 10.1016/s0300-9572(97)00089-0 9547841

[B38] Pell JP Sirel JM Marsden AK Ford I Walker NL Cobbe SM Presentation, management, and outcome of out of hospital cardiopulmonary arrest: comparison by underlying aetiology Heart 2003 89 8 839 842 10.1136/heart.89.8.839 12860852 PMC1767789

[B39] Castren M Kurola J Nurmi J Martikainen M Vuori A Silfvast T Time matters; what is the time in your defibrillator? An observational study in 30 emergency medical service systems Resuscitation 2005 64 3 293 295 10.1016/j.resuscitation.2004.08.017 15733756

